# Biomimetic Silk Architectures Outperform Animal Horns in Strength and Toughness

**DOI:** 10.1002/advs.202303058

**Published:** 2023-08-18

**Authors:** Yawen Liu, Yushu Li, Qiyue Wang, Jing Ren, Chao Ye, Fangyuan Li, Shengjie Ling, Yilun Liu, Daishun Ling

**Affiliations:** ^1^ Frontiers Science Center for Transformative Molecules School of Chemistry and Chemical Engineering State Key Laboratory of Oncogenes and Related Genes National Center for Translational Medicine Shanghai Jiao Tong University Shanghai 200240 China; ^2^ School of Physical Science and Technology ShanghaiTech University 393 Middle Huaxia Road Shanghai 201210 China; ^3^ Laboratory for Multiscale Mechanics and Medical Science SV LAB School of Aerospace Xi'an Jiaotong University Xi'an 710049 China; ^4^ Shanghai Clinical Research and Trial Center Shanghai 201210 China; ^5^ World Laureates Association (WLA) Laboratories Shanghai 201203 China

**Keywords:** biomaterials, biomimetic silks, Light, strong, and tough materials (LSTMs), mesostructures, textile processing

## Abstract

Structural biomimicry is an intelligent approach for developing lightweight, strong, and tough materials (LSTMs). Current fabrication technologies, such as 3D printing and two‐photon lithography often face challenges in constructing complex interlaced structures, such as the sinusoidal crossed herringbone structure that contributes to the ultrahigh strength and fracture toughness of the dactyl club of peacock mantis shrimps. Herein, bioinspired LSTMs with laminated or herringbone structures is reported, by combining textile processing and silk fiber “welding” techniques. The resulting biomimetic silk LSTMs (BS‐LSTMs) exhibit a remarkable combination of lightweight with a density of 0.6–0.9 g cm^−3^, while also being 1.5 times stronger and 16 times more durable than animal horns. These findings demonstrate that BS‐LSTMs are among the toughest natural materials made from silk proteins. Finite element simulations further reveal that the fortification and hardening of BS‐LSTMs arise primarily from the hierarchical organization of silk fibers and mechanically transferable meso‐interfaces. This study highlights the rational, cost‐effective, controllable mesostructure, and transferable strategy of integrating textile processing and fiber “welding” techniques for the fabrication of BS‐LSTMs with advantageous structural and mechanical properties. These findings have significant implications for a wide range of applications in biomedicine, mechanical engineering, intelligent textiles, aerospace industries, and beyond.

## Graphical abstract

Inspired by the intricate interwoven mesostructures of peacock mantis shrimp, the biomimetic silk LSTMs (BS‐LSTMs) are designed and fabricated via combined textile processing techniques with fiber “welding”. The resulting BS‐LSTMs exhibit a remarkable combination of lightweight (density of 0.6–0.9 g cm^−3^) and strength (1.5 times stronger) and durability (16 times more durable) than animal horns.

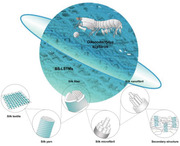

## 1 Introduction

Light, strong, and tough materials (LSTMs) are highly demanded in a variety of fields, including mechanical engineering,^[^ ^1^, ^2^, ^3^, ^4^, ^5^ ^]^ biomedicine,^[^ ^6^, ^7^ ^]^ intelligent textiles,^[^ ^8^, ^9^, ^10^, ^11^ ^]^ and aerospace industries.^[^ ^7^ ^]^ However, constructing high‐performance LSTMs is still a significant challenge in material engineering because of the inherent conflict between mechanical and structural properties.^[^ ^1^, ^2^, ^3^ ^]^ For instance, inorganic materials (e.g., glasses, ceramic, diamond, and minerals) with high strength‐to‐weight (i.e., specific strength) and stiffness‐to‐weight (i.e., specific modulus) ratios are typically brittle and unsuitable for strong‐and‐lightweight applications, while lightweight and tough organic materials (e.g., rubbers) are often soft and easily deformable.^[^ ^12^ ^]^

Biological systems require lightweight, strong, and resilient structures.^[^ ^13^, ^14^ ^]^ Nature has developed mesostructures in biological LSTMs to satisfy diverse mechanical and functional requirements using limited and unremarkable starting materials. Even relatively weak components, such as proteins and polysaccharides are utilized to provide diversity in chemistry and biophysical interactions.^[^ ^15^ ^]^ Spider dragline silk and silkworm cocoon silks are composed of proteins;^[^ ^16^ ^]^ hairs, feathers, horns, and quills are made of keratin;^[^ ^17^, ^18^, ^19^ ^]^ and sucker ring teeth are entirely composed of silk‐like proteins.^[^ ^20^, ^21^ ^]^ Over billions of years of evolution, nature has developed sophisticated mesostructures at the mesoscale to meet the challenges of robust material design.^[^ ^22^, ^23^ ^]^ Many biological LSTMs share common meso‐construction characteristics, such as helicoidal structures, gradient mesoporous structures, and “brick‐and‐mortar” structures.^[^ ^14^, ^24^, ^25^ ^]^ These mesostructures impart materials with significantly increased toughness and fracture resistance, allowing for the rational design of materials with various functions, including structural support, camouflage, hunting, seed dispersion, sensing, and so on.^[^ ^26^, ^27^, ^28^, ^29^, ^30^, ^31^ ^]^

Biomimetic designs inspired by natural mesoarchitectures have shown remarkable potential in materials science, from novel composites to the creation of sound through the translation of protein material structures.^[^ ^15^, ^26^, ^32^, ^33^ ^]^ Advanced processing techniques, including 3D printing, two‐photon lithography, and atomic layer deposition, have been used to fabricate bioinspired mesoarchitectures, such as helicoidal and lattice‐like structures with appealing properties for biomedical applications.^[^ ^15^, ^34^, ^35^, ^36^, ^37^, ^38^, ^39^, ^40^, ^41^, ^42^, ^43^, ^44^ ^]^ However, these methods are time‐consuming, and energy‐intensive, limiting their scalability and hindering the fabrication of complex structures, such as the sinusoidal crossed herringbone design of the dactyl club of the peacock mantis shrimp (Odontodactylus scyllarus), which remains a major challenge.^[^ ^45^, ^46^, ^47^, ^48^, ^49^ ^]^

**Scheme 1:**
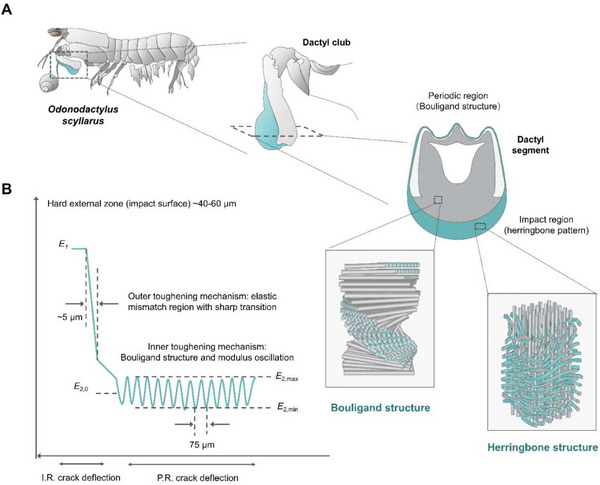
Structure‐mechanics relationship of the dactyl club of *Odonodactylus scyllarus*. A) Schematic of dactyl club separated from *O. scyllarus*. B) Toughening strategies of the dactyl club (middle): i) hard outer layer for maximum impact force; ii) modulus transitional region for crack deflection between the impact surface and the bulk of the impact region; iii) periodic region with helicoidal pattern and modulus oscillation for crack shielding. *E*, electric field vector. Adapted with permission from the American Association for the Advancement of Science (AAAS)^[^ ^44^ ^]^ and John Wiley and Sons.^[^ ^47^ ^]^

The impressive mechanical performance of the dactyl club of the peacock mantis shrimp, capable of delivering strikes with accelerations exceeding 105 m s^−2^ and speeds of 23 m s^−1^, is due to its superior gradient mesoarchitectures consisting of the impact and periodic regions (**Scheme** **1**).^[^ ^46^, ^47^, ^48^, ^49^, ^50^ ^]^ The impact region features a compact and pitch‐graded sinusoidal arrangement of helicoidally arranged mineralized nanofibrils that enhance stress redistribution and out‐of‐plane stiffness, while sinusoidal interfaces in the herringbone structure extend the crack propagation path.^[^ ^49^ ^]^ The periodic region is composed of partially mineralized chitin nanofibril and a helicoidal structure that dissipates energy through microcrack propagation, resulting in enhanced work of fracture.

Textile fabrication technologies offer unique advantages in constructing complex 3D intertwined mesostructures with precise interlacing and arrangement.^[^ ^51^, ^52^, ^53^, ^54^ ^]^ The vast number of texture pattern databases and manufacturing techniques developed over millennia of textile production, including plain weaves, loop configurations, and nonwoven techniques, can be utilized to create complex bioinspired and biomimetic 3D structures.^[^ ^55^, ^56^, ^57^, ^58^, ^59^, ^60^, ^61^ ^]^

Inspired by the intricate interwoven mesostructures of peacock mantis shrimp, we herein report on the designed fabrication of biomimetic silk LSTMs (BS‐LSTMs) via combined textile processing techniques with fiber “welding”. To create predesigned 3D textures that mimic natural mesostructures, silk fabrics were chosen as the architecture unit due to their unique structural hierarchy. Textile processing techniques were then employed to transform the textiles with loose fibers into a congealed and continuous network. This was achieved through a process called fiber “welding” which selectively etches the surface of the fibers, enabling the controllable regulation of meso‐interfaces via partial dissolution. The fiber retains its original configuration, while only its surface is dissolved, leaving behind dissolved polymers that serve as “welding” materials or adhesives to join the fibers. The resulting interfacial bondings between fibers reinforce the loose fabric, enabling all fibers to function as a mechanical load‐bearing unit that can transfer loads and regulate the mechanical behavior of the materials. These bioinspired BS‐LSTMs demonstrate exceptional mechanical properties, including lightweightness, high strength, and remarkable toughness, which are comparable to those of natural materials. Our work highlights the potential of integrating textile processing and fiber “welding” techniques for designing and fabricating LSTMs with advantageous structural and mechanical properties.

## 2 Results

### 2.1 Materials Selection of BS‐LSTMs

**Figure 1:**
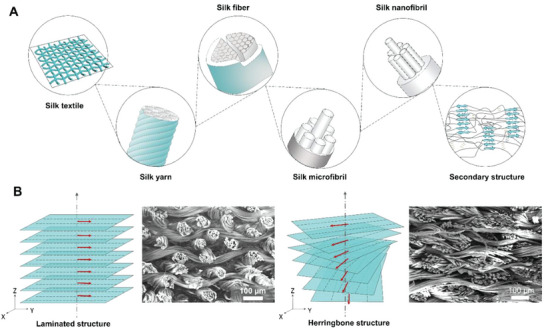
Preparation and Hierarchical Structural characterization of BS‐LSTMs. A) Schematic of the hierarchical structure of silk fabric. B) Illustrations of the stacking of silk fabric in BS‐LSTMs and SEM images of BS‐LSTMs.

The selection of silk fabrics for the fabrication of bioinspired mesostructures in this study was based on a multitude of compelling reasons. First, silk is one of the most ancient fabrics, and as such, it possesses a well‐established texture pattern and textile techniques that can serve as a reference for the construction of complex structures.^[^ ^62^ ^]^ Second, silks, which are protein fibers obtained from silkworm cocoons, are an abundant and sustainable material that can be utilized in numerous emerging fields, including biomedicine, structural engineering, and space‐related applications, owing to their inherent biocompatibility.^[^ ^63^ ^]^ Third, silks possess a unique structural hierarchy (**Figure** **1**A), which imparts superior strength and durability compared to most polymer fibers and confers greater tolerance toward flaws.^[^ ^23^ ^]^ Finally, our screening experiments have confirmed that silk fibers can be successfully “welded” using a variety of solvent systems, such as hexafluoroisopropanol (HFIP), ionic liquids, and formic acid/calcium chloride. Collectively, these characteristics make silk a highly attractive material for the fabrication of intricate bioinspired structures with diverse applications.

### 2.2 Design and Fabrication of BS‐LSTMs

The biomimetic silk LSTMs, or BS‐LSTMs for brevity, were inspired by the laminated and herringbone structures found in nature, as depicted in Figure 1B and Figure S1 (Supporting Information).^[^ ^14^, ^15^ ^]^ To create these structures along the *Z*‐axis of the 3D constructs, silk plain fabrics with a warp‐weft density of 5 × 5 threads per mm^2^ were stacked, with each layer rotated by a constant angle, *θ*, relative to the previous layer, depending on the spatial geometry of the configuration. The herringbone structure had a *θ* of ≈18°, while the laminated structure had a *θ* of 0. After stacking the fabrics, the next step was to fix them by knitting the silk threads along the *Z*‐axis with an embroidery machine, with a distance of 500 µm between adjacent yarns. It is worth noting that silk fabrics with a relatively lower warp‐weft density were selected for all structures, as the spaces between the warp and weft‐silk yarns can serve as solvent penetration channels for the “welding” of fibers.

**Figure 2:**
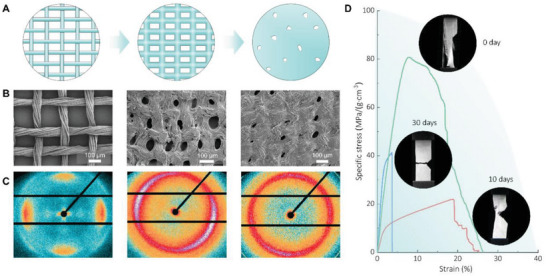
Structural changes during “welding” processing of silk fabric. A) Schematic of “welding” processing of silk fabric. B) SEM images of silk fabric after “welding” processing for 2, 10, and 30 days, respectively. C) WAXS patterns of silk fabric after “welding” processing for 0, 10, and 30 days, respectively. D) The representative stress–strain curves of silk fabric after “welding” processing for 0, 10, and 30 days, respectively. The insert photographs show the crack propagation processes of silk fabric after “welding” processing.

Despite their biomimetic design, these BS‐LSTMs lacked sufficient mechanical strength due to the limited interplay between the loose silk yarns in all directions, resulting in a lack of 3D bulk formation (Figure S2A,B, Supporting Information). To address this, fiber “welding” (Figure S2C, Supporting Information) was performed to reinforce the structures. The 3D silk fabric was immersed in a hexafluoroisopropanol (HFIP) solution and incubated for 2–30 days at 60 °C. Referring to our previous work,^[^ ^64^, ^65^ ^]^ during incubation at 60 °C, the HFIP gradually permeated into the silk fibers from the defects and ends and partially dissolved the sheath layer into silk fibroin polymer chains. Due to the limited dissolution effect of HFIP, the silk fibers are partially dissolved, and the dissolution degree increase with the prolongation of dissolution time. Then, HFIP is volatile and the dissolved silk fibers were adhesive and could act as glue to bind the fibers together, strengthening the fabric. HFIP penetrated the fabric and partially dissolved the surface of the silk fibers (**Figure** **2**A), as confirmed by scanning electron microscopy (SEM) (Figure 2B), wide‐angle X‐ray scattering (WAXS) (Figure 2C; Figure S3, Supporting Information), and polarizing microscopy (Figure S4, Supporting Information). The dissolved silk fibroin fibers were adhesive and could act as glue to bind the fibers together, strengthening the fabric. Fourier transform infrared spectroscopy (FTIR) (Figure S5A,B, Supporting Information) confirmed that the dissolved silk fibroin could reform into *β*‐sheets after drying at 60 °C, acting as cross‐linkers to form interlocking protein chains that strengthened the bonding of the fibers and yarns. The deconvolution of the amide I band in Figure S5C (Supporting Information) further revealed the secondary structure content of the silk fibers, with *β*‐sheet, random coil/helix, and *β*‐turn accounting for 21%, 70%, and 9%, respectively. Additionally, the FTIR spectra of silk fabrics with fiber “welding” for different durations (Figure S6, Supporting Information) indicated that there were no significant changes observed in the secondary structures of the silk fibers after the “welding” process. In Figure 2C and Figure S3 (Supporting Information), it can be observed that the silk fibers maintain their characteristic *β*‐sheet structure even after the welding treatment. There is no apparent phase change in the structure. However, the 010 reflection is significantly reduced, and the intensity distributions in the circumference direction become broader as the incubation time in HFIP solution is prolonged. This is attributed to the partial dissolution of the silk fibers during the incubation process. As a result, the crystallite orientation of the dissolved silk fibers decreases as illustrated in Figure S3B (Supporting Information). After 30 days of incubation, the tensile failure mode of a 2D silk fabric changed gradually from tear mode to bulk fracture mode (Figure 2D). In comparison to the silk fabric without “welding” treatment, the load‐bearing capacity of the welded silk fabric does not exhibit significant improvement under tension. However, it is evident that the mechanical properties of the silk fabric improve with prolonged incubation time. This can be attributed to the fact that the fibers of the silk fabric are relatively loose prior to the welding treatment, so it is difficult to form stress concentration at the tip of defect. After undergoing the “welding” treatment, the resulting interfacial bondings enable the silk fabric to function as a mechanical load‐bearing unit capable of effectively transferring loads.

**Figure 3:**
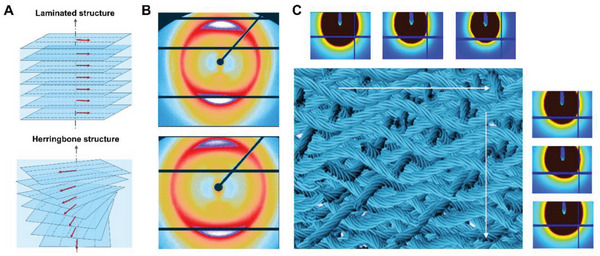
Structural characterization of BS‐LSTMs. A) Schematics of stacking structures of BS‐LSTMs after “welding” processing. B) WAXS patterns of BS‐LSTMs after “welding” processing for 10 days. C) Synchrotron SAXS patterns of BS‐LSTMs after “welding” processing for 10 days.

**Figure** **3** and Figures S7–S9 (Supporting Information) depict the mesostructures of the BS‐LSTMs following fiber “welding”. The BS‐LSTMs had a density of between 0.6 and 0.9 g cm^−3^ (**Table** **1**). The integrated bulk system is illustrated using color transitions from white to yellow (Figure S7, Supporting Information). This choice is made because the dissolved liquid crystal of silk, which is partially dissolved by HFIP, exhibits a yellow color. Thus, the degree of dissolution can be inferred from the intensity of yellow coloration, with a higher degree of dissolution resulting in a more pronounced yellow hue. ^[^ ^64^, ^65^ ^]^ The predesigned mesostructures were preserved, as shown by WAXS (Figure 3B; Figure S8, Supporting Information) and SEM (Figure S9, Supporting Information). In Figure S8 (Supporting Information), it is evident that the BS‐LSTMs with welding treatment, whether they have a laminated or herringbone structure, exhibit no significant phase change compared to those without treatment. Additionally, the azimuthal WAXS profiles of the BS‐LSTMs after the “welding” process indicate that the crystallite orientation of the dissolved silk fiber also inclines toward the fiber axis. This finding supports the observation of the crystallite orientation change in the dissolved silk fibers, reinforcing the notion that the welding treatment does not induce a noticeable phase change in the structure of the BS‐LSTMs. The SEM images in Figure S10 (Supporting Information) demonstrate that the degree of fiber “welding” increased gradually as the incubation time in HFIP solution was prolonged. Consequently, the adhesion between the fibers became more pronounced, leading to a reduction in the size of the BS‐LSTMs. Additionally, the pores present in the silk fabric showed a decrease in size as a result of the “welding” process. High spatial resolution synchrotron small angle X‐ray scattering (SAXS) was used to examine the detailed mesostructures of herringbone features, a continuous scan mode with a step‐length of 50 µm was used to examine the alignment of silk fibers in the cross‐sectional plane (Figure 3C). The resulted SAXS patterns revealed the differentiated orientation of fiber framework in both the *Y* and *Z* directions, consistent with the mesostructure modifications in the herringbone architecture. Overall, these results demonstrate the successful reinforcement of the BS‐LSTMs, resulting in intricate and robust bioinspired mesostructures with potential applications in various fields.

**Table 1:** Density and mechanical properties of BS‐LSTMs with different welding periods

BS‐LSTMs	Welding periods [days]	Density [g cm^−3^]	Mechanical properties		
			Specific strength [MPa (g cm^−3^)^−1^]	Specific stiffness [MPa (g cm^−3^)^−1^]	Specific toughness [MPa (g cm^−3^)^−1^]
Laminated structure	2	0.78 ± 0.03	34.9 ± 5.5	608.1 ± 59.8	8.7 ± 0.7
	10	0.66 ± 0.05	35.0 ± 2.3	374.4 ± 23.6	8.3 ± 1.1
	20	0.78 ± 0.12	35.48 ± 5.5	376.7 ± 22.1	8.1 ± 0.7
	30	0.76 ± 0.17	34.5 ± 2.9	338.3 ± 64.8	8.6 ± 0.1
Herringbone structure	2	0.69 ± 0.06	33.1 ± 1.6	485.1 ± 95.3	9.3 ± 2.0
	10	0.82 ± 0.01	33.6 ± 1.8	548.3 ± 13.3	9.9 ± 0.3
	20	0.82 ± 0.02	30.9 ± 2.2	476.4 ± 74.7	8.7 ± 1.3
	30	0.74 ± 0.06	36.9 ± 1.6	740.8 ± 32.7	9.2 ± 0.2
Buffalo horn		1.12 ± 0.12	24.1 ± 2.6	659.4 ± 105.5	0.59 ± 0.17

### 2.3 Mechanical Performance of BS‐LSTMs

**Figure 4:**
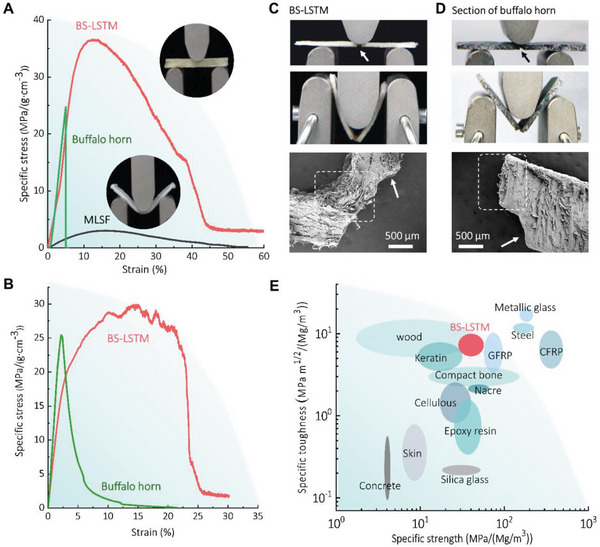
Mechanical performance of BS‐LSTMs. A) The representative stress–strain curves of BS‐LSTM, multilayer silk fabric (MLSF) and Buffalo horn. B) The representative stress–strain curves of notched BS‐LSTM and notched section of Buffalo horn. C) Photograph of three‐bending tests of notched BS‐LSTM. SEM image at the bottom is the fracture surfaces of notched BS‐LSTM. D) Photograph of three‐bending tests of notched Buffalo horn. SEM image at the bottom is the fracture surfaces of the notched Buffalo horn. E) Comparison of specific strength and specific toughness of BS‐LSTMs with other materials. The Ashby plot was drawn according to the data extracted from refs. [54, 55, 56].

The mechanical properties of BS‐LSTMs were evaluated using three‐point bending tests, and representative stress–strain curves display graceful deformation without abrupt collapse (**Figure** **4**A). This characteristic exhibits the same compression behavior as hard biological materials, such as cancellous bone and wood.^[^ ^23^ ^]^ At small strains, the behavior is elastic, but beyond the elastic regime, the loading curves exhibit a large and gradual descent zone. Moreover, these 3D architectures exhibit a remarkable balance between strength and toughness. Indeed, the “welding” process plays a crucial role in enhancing the interaction between the layers and fibers within the BS‐LSTMs. This enhancement leads to a substantial improvement in the load‐bearing capacity of the material under shear and compression. As a result, the mechanical properties of the BS‐LSTMs are significantly enhanced. The strengthened bonding between the layers and fibers enables the material to better distribute and withstand applied forces, resulting in improved overall mechanical performance. Herringbone structures, whose specific strength (σ_f_/ρ) reached 36.9  ± 1.6 MPa (g cm^−3^)^−1^, were stronger than non‐welded 3D silk fabrics (2.8 ± 0.2 MPa (g cm^−3^)^−1^). This particular flexural strength is 1.5 times that of buffalo horn, one of the hardest biological protein materials (Table 1).^[^ ^17^ ^]^ Intriguingly, no direct relationships were found between welding periods and mechanical properties (Figure S11, Supporting Information), indicating that 2 days of welding were sufficient to produce BS‐LSTMs. In contrast, the specific toughness was more than 16 times greater than that of buffalo horn. Therefore, the welding process is essential for fabricating BS‐LSTMs with remarkable strength and fracture toughness.

### 2.4 Fracture Behavior of BS‐LSTMs

Single‐edge notched bending (SENB) was utilized in conjunction with an ultrahigh‐speed camera system to determine the fracture toughness of the silk fabric. Laser cutting was utilized to create an artificial notch of the desired width in the silk fabric. The stress–strain curves of notched silk fabrics and buffalo horn are depicted in Figure 4B. When the notch was smaller than half the width of the fabric (as indicated by the arrows in Figure 4C,D), the notched fabric exhibited the same stress–strain curves as the unnotched samples; only the specific strength was reduced (from 37 to 30 MPa (g cm^−3^)^−1^), exhibiting typical ductile fracture behavior. Buffalo horn, on the other hand, exhibited brittle failure behavior and linear crack propagation in the notched sections that fractured before reaching the yield point (a breaking strain of 2%–7%, Figure 4B; Figure S12, Supporting Information). The specific fracture toughness (*K*_JC_/ρ) of BS‐LSTMs was then calculated using the *J*_R_ method.^[^ ^66^, ^67^, ^68^, ^69^, ^70^ ^]^ The specific fracture toughness reached 7.2±1.2 MPa m^1/2^ (Mg m^−3^)^−1^, a value 1.4–3.8 times that of Cristaria plicata nacre (2.2–2.4 MPa m^1/2^ (Mg m^−3^)^−1^) and compact bone (2.2–2.4 MPa m^1/2^ (Mg m^−3^)^−1^), and even superior to the majority of other natural and engineered materials (Figure 4E).^[^ ^71^, ^72^, ^73^ ^]^

### 2.5 Physical Mechanisms of BS‐LSTMs

To further investigate the underlying deformation and hardening mechanisms, we created simulations of BS‐LSTMs at multiple scales using the finite element method (FEM). In the Supporting Information, the setup and loading conditions of the model are described in detail. In total, four models were developed to examine the gradual “welding” effects observed in experiments. These models, labeled “Weld 0”, “Weld 1”, “Weld 2”, and “Weld 3”, refer to the gradual increase in welding degree (from no welding to significant welding) that occurred after longer incubation in HFIP solution (Figure S13, Supporting Information).

**Figure 5:**
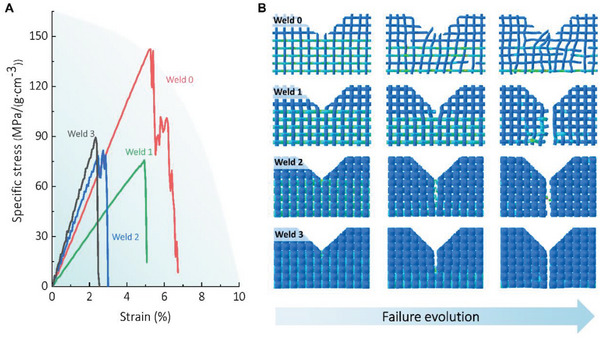
FEM simulations of silk fabric. A) Stress–strain curves of the silk fabric models at different degrees of “welding” processing subject to tensile loading. B) Simulation snapshots of the stress contribution and failure evolution within the silk fabric under different degrees of “welding” processing.

As depicted in **Figure** **5**A, the silk fabric model was initially subjected to uniaxial tensile loading, with a notch created on one side of the fabric to promote fracture. The recorded stress–strain curves demonstrate consistent trends with the experiments (Figure 2D): the fabric's behavior becomes more brittle as the welding exerts greater effects. The simulation snapshots revealed the failure progression of each model under varying welding intensities (Figure 5B). In the absence of welding, the fabric did not respond to the predetermined notch. As the fabric was subjected to a tensile load, the fabric's threads nearly deformed independently due to the lack of binding between the fibers. According to the snapshots, no obvious stress concentration was detected. The response can be viewed as the average response of the fibers that comprise the material. Consequently, the failure of the fabric was also determined by each fiber separately: fibers with higher stress failed before those with lower stress. This behavior explains why the failure of the fabric does not begin at the tip of the notch and progress to the opposite side, as was observed in experiments. As welding was introduced, the silk fibers became intertwined, and a stress concentration was observed at the notch's tip, with the degree of stress concentration increasing with the degree of welding. Consequently, the thread at the crack tip reaches its strength limit earlier than other threads, and each failure of the thread at the tip exposes the next thread to the crack front, causing it to fail sooner than the others. Eventually, the fabric fails due to a series of thread failure cycles. This leaves a clearer crack for a higher degree of welding, following experimental findings (Figure 2D). Due to the disparity in properties between the fibers and the welding interface, interfacial welding acts as a soft glue to bind the fibers during lower‐degree welding, leaving the fibers connected after the failure of the fabric. This result suggests that there would be an optimal degree of welding to maximize the benefits of toughening the material: below which the threads deform more independently and the material behaves softer, and above which, although the material would behave stiffer, the properties discrepancy between threads and interfaces tends to diminish and the material behaves brittle. The simulation results did not demonstrate a close alignment with the experimental data, which could be attributed to several factors. One possible reason is the limited dissolution effect of HFIP during the experimental process, which may not be accurately captured in the simulation. Additionally, it can be challenging to establish a precise correspondence between the welding period in the experimental setup and the welding degree in the simulation. Considering that longer incubation periods lead to an increase in the degree of dissolution, it is reasonable to assume that the integrity of silk materials may decrease and potentially result in a reduction in their mechanical properties. This hypothesis aligns with the notion that as the dissolution degree increases, the bonding between fibers and the overall structure may be compromised, leading to a potential decrease in mechanical performance. It is important to note that welding does not increase the load‐bearing capacity in tension, but it does improve the load‐bearing capacity in shear, compression, and other modes. As a result, welding is a critical process for enhancing the performance of BS‐LSTMs under complex loads.

**Figure 6:**
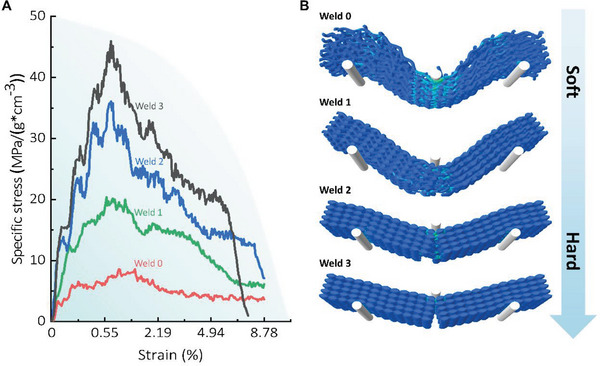
Three‐bending simulations of BS‐LSTMs. A) Stress–strain curves of BS‐LSTMs models with laminated structures at different degrees of welding subject to three‐bending tests. B) Simulation snapshots of the stress contribution and failure evolution within the BS‐LSTMs under different degrees of “welding” processing.

Similarly to tensile tests, welding has a substantial effect on the response of BS‐LSTMs with a laminated structure (model details in Supporting Information). Consistent with experimental findings, the flexural stress–strain curves in **Figure** **6** reveal that material with a lower degree of welding behaves softer, while material with a higher degree of welding behaves brittle. A closer examination of the deformation and crack evolution of the model with a lower degree of welding (Figure 6B) reveals that broken threads still connected the material across the interface of the crack, and small thread segments were pulled out after the interfacial welding was broken. This is because the composed fibers are less constrained by their neighboring fibers, making it easier to relax the structure, resulting in the outer layers carrying the majority of the load. By introducing mechanisms of energy dissipation and crack path deflection, appropriate welding contributes to the toughness of the material, as demonstrated by this finding. In the case of the highest degree of welding, the material failed after the tension side's strength limit was reached. The material is being broken apart by a crack that began at the bottom. The formation of a neat crack through the depth indicates further synergetic deformation.

**Figure 7:**
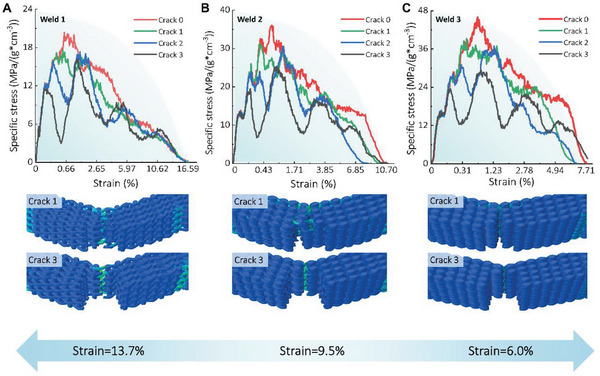
Simulations of BS‐LSTMs with artificial notches. A) Flexural stress–strain curves and snapshots of “Weld 1” BS‐LSTMs models with an artificial notch at different degrees of cracks in three bending tests. B) Flexural stress–strain curves and snapshots of “Weld 2” BS‐LSTMs models with an artificial notch at different degrees of cracks in three bending tests. C) Flexural stress–strain curves and snapshots of “Weld 3” BS‐LSTMs models with artificial notches at different degrees of cracks in three bending tests.

To further investigate this phenomenon, identical loads were applied to models with an artificial notch in a three‐point bending setup. As depicted in **Figure** **7**, BS‐LSTMs with lower welding are pliable, whereas those with higher welding are brittlely. The presence of a notch creates a discontinuity in the bulk and complicates the bending behavior of models. There are oscillations in the stress–strain curves. In contrast, the failure evolution is comparable to tensile tests. “Crack 0” to “Crack 3” indicate the increasing depth of the artificial notch used to validate the fracture mechanism. For materials with a lower degree of welding, cracks tend to originate from interconnected regions and exhibit an irregular crack path, whereas, for materials with a higher degree of welding, cracks are systematically located in the middle bottom region of models. Overall, the bending behaviors of the unnotched and notched models are comparable, and the pre‐set crack reduces the strength to some degree.

Furthermore, research has confirmed that composites with herringbone structures and helicoidal organization offer toughening capabilities through crack twisting and fiber bridging.^[^ ^74^, ^75^ ^]^ To compare the efficacy of different models, herringbone structures were incorporated into the analysis. Additional details can be found in the Figures S14 and S15 (Supporting Information). The mechanical behaviors of models with both laminate and herringbone structures were investigated, and similar results were observed.

## 3 Conclusion

In summary, our study demonstrates the successful integration of textile processing and fiber “welding” techniques to fabricate complex bioinspired 3D architectures with interlaced mesostructures. These unique features cannot be replicated using traditional or advanced materials processing methods. The scalability of textile technology and fiber “welding” processes makes them well‐suited for large‐scale production and industrial applications. One promising application for our biomimetic silk LSTMs is the development of artificial horns or horn substitutes, which could help combat the illegal trade of rhino and elephant horns. Our BS‐LSTMs are not only more robust than chemically cross‐linked animal horns, but also comparable to nacre and bone, two well‐known bionanomineral‐based strong and resilient materials. Moreover, our approach achieved mechanical reinforcement through structure designs instead of incorporating additional components, highlighting the potential of these techniques for the construction of other structural and functional LSTMs.

We anticipate that future research will explore the incorporation of functional components, such as conductive nanomaterials and photoelectric fillers during fiber “welding”, as well as the use of machine learning to predict and optimize the mechanical properties of these materials.^[^ ^76^, ^77^ ^]^ Overall, our study showcases a rational, cost‐effective, and efficient strategy for designing and fabricating LSTMs, with broad potential applications in biomedicine, mechanical engineering, intelligent textiles, aerospace industries, and beyond.

## 4 Experimental Section

### Fabrication of Biomimetic 3D Silk Structures

*Bombyx mori* (*B. mori*) silk fabrics were utilized to create three distinct 3D structures, including laminated structure, helicoidal structure, and herringbone. Silk plain fabrics with a warp‐weft density of 5 × 5 threads per mm^2^ were stacked in a sequence along the fabric's normal direction to create laminated and helicoidal structures (*Z*‐axis of the 3D constructions). Depending on the spatial geometry of each configuration, each layer of the fabric was rotated relative to the previous stacked layer by a constant angle, θ, during stacking. Specifically, the θ of a laminated structure was 0; that is, the warp and weft in different layers were aligned parallel to one another. In contrast, the θ of a helicoidal structure was ≈18°, allowing these plain silk fabrics to stack helicoidally in the normal direction of the fabric. The fabrics were then stitched using an automatic embroidery machine. With a yarn distance of 500 µm, the silk yarns were sewed along the *Z*‐axis in this procedure. In these structures, the spline areas of the laminated structure and the herringbone structure were regulated to be 50 × 50 mm.

### Silk “Welding” Processing

The biomimetic 3D silk structures were immersed in a 1:50 weight ratio hexafluoroisopropanol (HFIP) solution and incubated at 60 °C, the boiling point of HFIP, for 2 to 30 days. All procedures were carried out in a chemical fume hood using the necessary safety precautions. During this procedure, the HFIP partially dissolved the silk fibers, resulting in the formation of adhesive surfaces. The HFIP‐stabilized dissolvable silk proteins appeared as a uniform solution phase. The resulting 3D silk structures were then air‐dried. The residual HFIP could be eliminated by heating it to 70 °C or exposing it to the chemical hood for ≈1 week.

### X‐Ray Scattering Experiments

WAXS and SAXS were used to examine the structural organization of biomimetic 3D silk constructions. At the Characterization and Analysis Center of ShanghaiTech University, WAXS experiments were conducted using Xenocs WAXS equipment, Xeuss 2.0. The detector collected diffraction patterns with 619 × 487 pixels of 172 µm × 172 µm area each. The X‐ray source's wavelength and photon flux were 1.54189 Å, and 4.0 × 10^7^ photons s^−1^, respectively. At the detector, the beam dimensions were 1.2 × 1.2 mm. On the Shanghai synchrotron source beamline BL19U2 (Shanghai, China), SAXS experiments were conducted with a 1.03 Å wavelength and a high photon flux 1.03 Å, delivering a high‐photon flux (5 × 10^12^ photons s^−1^) on the sample. The diffraction patterns were gathered using a CMOS hybrid pixel detector with a total of 172 × 172 pixels. The horizontal beam size at the detector was fixed at 0.33 × 0.05 mm (vertical).

### Mechanical Tests

To examine the mechanical performance of the dried silk fabrics and BS‐LSTMs, both tensile and three‐bending tests were conducted. All tests were conducted at room temperature and relative humidity (RH) of 45% using Instron 5966 (Instron, Norwood, USA). For the tensile tests, 30 mm (length) by 10 mm (width) splines were utilized. The rate of tension was 2 mm min^−1^. The three‐point bending tests were conducted in flexural test mode at 25 °C and 45% RH with a 2 mm min^−1^ loading rate. Following the Instron 5966‐2810‐400 bend fixture, specimens measuring 44 mm (length) × 22 mm (width) × 1.2 mm (thickness) were utilized for all three bending tests, with the span‐to‐depth ratio set to 16. $\sigma_{f}=\frac{3PL}{2BD^{2}}\;$ was used to calculate the specimen's strength, and $\varepsilon_{f}=\frac{6Dd}{L^{2}}\;\;$was used to calculate its strain, where *P* represents the load, *D* represents the specimen's thickness, *B* represents the specimen's width, *d* represents beam deflection, and *L* represents the specimen's span. The area above the normal flexural specific stress–strain curve was the specific toughness. Notably, each stress–strain curve illustrated in this article was derived from a single test, but the data obtained from the curves were the mean and standard deviations of at least five separate tests. The notch was 1/3–1/2 of the thickness of the specimen for SENB testing.

### Characterization

Silk fabrics and biomimetic 3D silk structures were characterized morphologically using polarizing optical microscopy (Olympus BX51‐P, Japan) and high‐resolution SEM (JEOL JSM‐790F, Tokyo, Japan) at an acceleration voltage of 5 kV. Before observation, all samples were sprayed for 20 s with a layer of gold to impart conductivity for SEM measurements. Using a Bruker 66v/s FTIR spectrometer, FTIR spectra were recorded. 128 interferograms were added and transformed using a Genzel–Happ apodization function to produce spectra with a nominal resolution of 8 cm^−1^ for each measurement time.

### Methods for *J*_R_ Calculation

For SENB testing, fracture toughness (*K*_Jc_) was determined by adding the elastic and plastic contributions, which correspond to the *J*_R_ calculation.

(1) $$J_{\mathrm{sum}}=J_{\mathrm{el}}+J_{\mathrm{pl}}$$

*J*_el_ represents the elastic contribution based on linear elastic fracture mechanics. One of them

(2) $$J_{\mathrm{el}}=\frac{K_{\mathrm{IC}}^{2}}{E^{\prime }}$$

(3) $$K_{\mathrm{Ic}}=\frac{P_{\mathrm{Ic}}S}{BW^{\frac{3}{2}}}\;f\left(\frac{a}{W}\right)$$

(4) $$E^{\prime }=E\;\left(1-v^{2}\right)$$

(5) $$\begin{array}{c} f\;\left(\frac{a}{W}\right)=\frac{\begin{array}{c} 3\;{\left(\frac{a}{W}\right)}^{\frac{1}{2}}\left[1.99-\left(\frac{a}{W}\right)\left(1-\frac{a}{W}\right)\left(2.15-3.93\frac{a}{W}+{\left(\frac{a}{W}\right)}^{2}\right)\right]\\ \; \end{array}}{2\left(1+2\frac{a}{W}\right){\left(1-\frac{a}{W}\right)}^{\frac{3}{2}}} \end{array}$$

where *K*_Ic_ is the stress intensity factor, *E* is Young's modulus, *v* is Poisson's ratio, *P*_Ic_ is the maximum load before crack initiation, *S* is the support span, *B* is the width of the sample, and *w* is the thickness of the sample. The sample was cut into ≈20.0 × 2.0 × 1.5 mm^3^ and *a* is the notch depth (*a*/*w*  = 0.45–0.55).

*J*_pl_ is the plastic contribution part and could be calculated with the following equation:

(6) $$J_{\mathrm{pl}}=\frac{2A_{\mathrm{pl}}}{B\left(W-a\right)}$$

where *A*_pl_ is the plastic area underneath the load‐displacement curve.

Therefore, the value of *K* could be inversely calculated from *J*_sum_ by the following equation:

(7) $$K_{\mathrm{Jc}}={\left(J_{\mathrm{sum}}E{}^{\prime }\right)}^{\frac{1}{2}}$$

where *K*_JC_ is the stress intensity factor, which consists of the elastic and plastic contribution. Herein, *E*′ can be replaced by *E*, since the change in *E* has a limited effect on the change in *K*_Jc_.

### Finite Element Method Simulation

Utilizing the finite element method (FEM), the mechanical behavior of BS‐LSTMs was investigated. “TexGen” was used to construct the models, while “Abaqus” was used for the calculations. The goal of the simulations was to uncover the inner mechanism that benefits the design of materials by reducing experimental costs and revealing subtle design rules. Since it was difficult to create high‐fidelity simulations that were identical to experiments, the critical factors that dominate simulation results were taken into account. The simulation's specifics are presented in the Supporting Information.

## Conflict of Interest

The authors declare no conflict of interest.

## Supporting information

Supporting Information

## Data Availability

The data that support the findings of this study are available from the corresponding author upon reasonable request.
