# Supplementary material for: Biomimetic Silk Architectures Outperform Animal Horns in Strength and Toughness
Source: Adv Sci (Weinh). 2023 Aug 18;10(29):2303058. doi: 10.1002/advs.202303058 (PMC10582412; doi:10.1002/advs.202303058)
Supplement: Supplementary file 1 — Supporting Information [file ADVS-10-2303058-s001.pdf]

ADVANCED SCIENCE
Open Access

Supporting Information

for *Adv. Sci.*, DOI 10.1002/advs.202303058

Biomimetic Silk Architectures Outperform Animal Horns in Strength and Toughness

Yawen Liu, Yushu Li, Qiyue Wang, Jing Ren, Chao Ye, Fangyuan Li, Shengjie Ling, Yilun Liu* and Daishun Ling**

Supporting Information

Biomimetic Silk Architectures Outperform Animal Horns in Strength and Toughness

Yawen Liu, Yushu Li, Qiyue Wang, Jing Ren, Chao Ye, Fangyuan Li, Shengjie Ling, Yilun Liu*, and Daishun Ling**

Supporting information contains:

Supplementary FEM simulation methods

Figures S1 to S13

Tables S1 to S2

Supplementary References

Supplementary FEM simulation methods

Model: The high-fidelity models were generated using the TexGen software, with the geometric parameters for tensile and three-point bending models presented in **Figure S13A**, **13B**, and **13E**. The cross-sectional shape of the threads without welding was observed to be circular with a diameter of 65 μm (**Figure S1**). However, welding was found to enlarge the volume of the threads, leading to a change in cross-sectional shape from a circle to an ellipse while the short axial dimension remained unchanged due to no significant increase in thickness during the experiment. The different degrees of welding is illustrated in **Figure S13C**, showing the variations in the thread's cross-sectional shape. Additionally, the welding process results in the connection of adjacent threads, and this is represented in the simulations by merging the FEM nodes together, as demonstrated in **Figure S13D**. The models with higher degrees of welding exhibit stronger connections.

To optimize computational efficiency, the sizes of the models used for tensile and three-point bending tests were simplified. For the bending models with laminate structures, the span-to-depth ratio was set at 4.9. Additionally, three pre-set crack cases were specified in the bending models: crack 1 with a thickness of 0.13 mm, crack 2 with a thickness of 0.26 mm, and crack 3 with a thickness of 0.39 mm. The cracks were located in the middle of the bottom of models with a length of 0.4 mm and a depth of 1.6 mm, as shown in **Figure S13E**. The cracks were implemented by deleting the corresponding FEM elements. The study also includes an analysis of three-bending models with a herringbone structure, with further details provided below.

The commercial software Abaqus was utilized for the simulations, with displacement loadings set for the tensile and three-bending tests. The C3D4 element was employed, and explicit dynamic analysis was specified.

Material: Uniaxial tensile experiments were conducted to determine the mechanical behavior of the thread in the longitudinal direction, also known as the 1-direction in the material coordinate system. The modules E_1 and tension strength X_T were found to be 2.5 GPa and 280 MPa, respectively. Other material properties used for the simulations were inferred by comparing them with those of carbon fiber yards.[1] The mechanical behaviors of the thread in both tensile and three-bending cases are determined by the properties in the 1-direction. The properties of welded materials in the 1-direction are obtained by scaling the cross-sectional area based on the materials of "weld 0" and "weld 1". The material parameters used in the simulations are listed in **Table S1**.

To indicate the failure of threads, the tension failure mode in the 1-direction is considered. The maximum stress failure theory is introduced,[2] which is expressed as:

$$I_F = \max\left(\frac{\sigma_{11}}{X_T}\right) < 1.0, \qquad \sigma_{11} > 0$$

$I_F = 1$ indicates that damage has occurred in the corresponding element under the underlying stress state and, as a result, has lost its loading capacity.

Homogenization of models with herringbone structure: The complex interactions between threads in different layers make it challenging to simulate three-bending models with herringbone structures. As a result, the homogenization method was employed to simplify these models, as demonstrated in **Figure S14**. Initially, a representative volume element (RVE) model was selected from the high-fidelity model (**Figure S14B**), and FEM is used to calculate the homogenization mechanical parameters of the RVE with periodic boundary conditions specified (**Figure S14C**).[3] Eventually, the homogenization model is constructed to represent the high-fidelity model, as illustrated in **Figure S14D**. Consequently, for three-bending models with herringbone structures, homogenization models are developed to simulate the mechanical behaviors (**Figure S14E**), while the geometric parameters remain unchanged as shown in **Figure S13**. It is worth noting that the RVE model comprises threads and matrix, and the material properties of the matrix are $E = 1, \upsilon = 0.3$, while the materials for threads are specified in **Table S1**. The calculated homogenization material parameters at different welding degrees are presented in **Table S2**. Modules E_1 and strength X_T increased with an increase in welding degrees, consistent with experimental results. Furthermore, materials with higher welding degrees tend to be more brittle, as can be inferred from the failure strain ε_{X_T}.

Additionally, both the 1-direction and 2-direction of the simplified materials are simultaneously considered.

Notably, the homogenization properties of the RVE models were acquired by averaging thread performance. Stress-strain curves for models with herringbone structures at different welding degrees were plotted in **Figure S15** for three-bending cases, including a comparison with the unnotched homogenization model with laminate structure under "Weld 2". The results indicate that both strength and stiffness increase with higher welding degrees, while pre-set cracks diminish the loading capacity of structures. Furthermore, the pre-set cracks lower the local span-to-width ratio, leading to an increase in failure strain, as illustrated in **Figure S15C**, **15D**, and **15E**.

It is also found that the unnotched model with laminate structure has a better performance in three-bending loading. Besides, failures in both 1-direction and 2-direction were observed for models with herringbone structure under "Weld 2", as displayed in **Figure S15B**. Overall, the welding process also has a significant influence on the models with herringbone structures. An appropriate welding degree can be chosen to balance the strength and stiffness in applications.

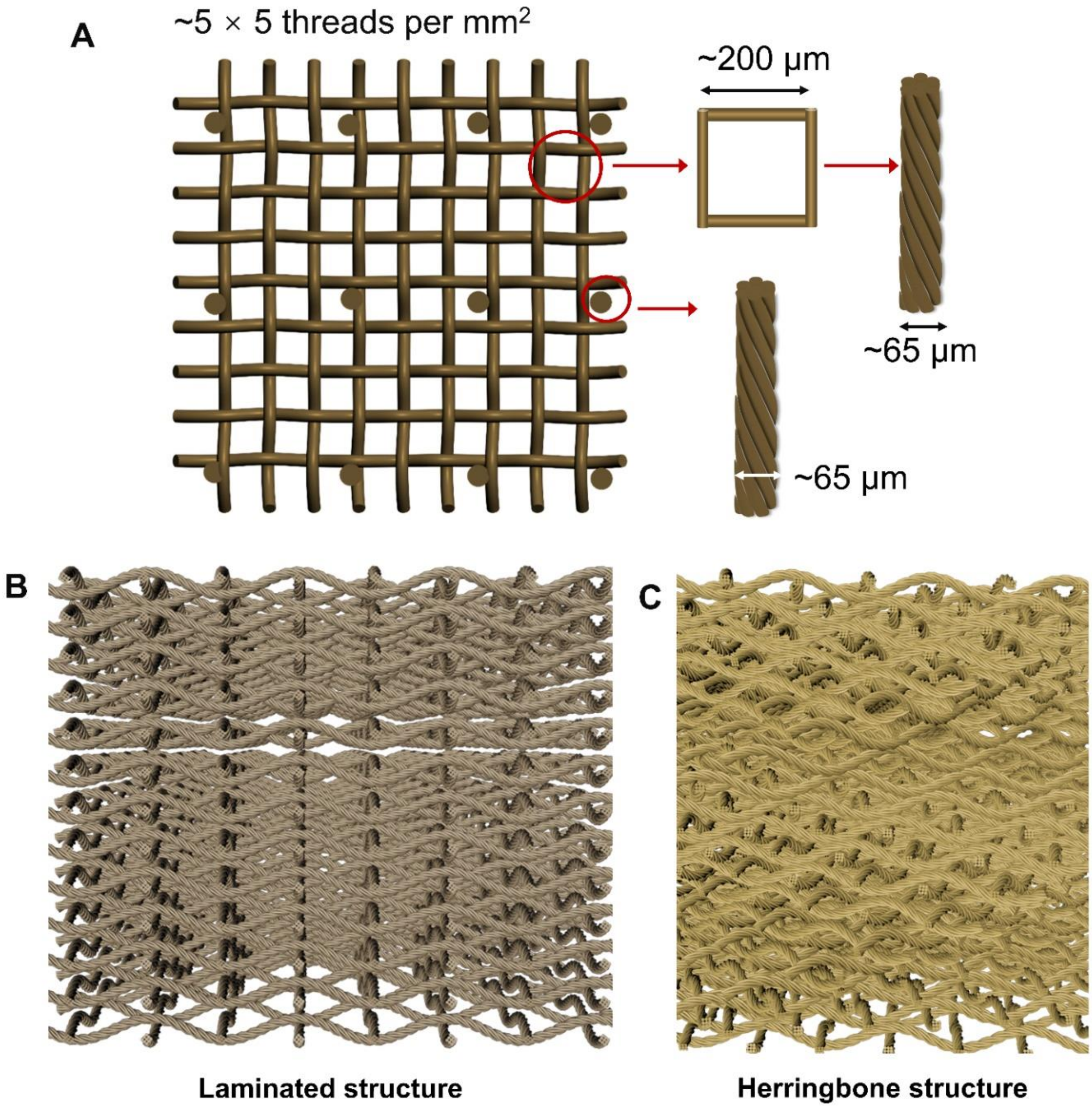

Figure S1. Geometrical structures of silk fabric and BS-LSTMs. A) Schematic of the geometrical structure of silk fabric used for constructing BS-LSTMs. B) Illustrations of the stacking of silk fabric in laminated structure. C) Illustrations of the stacking of silk fabric in herringbone structure.

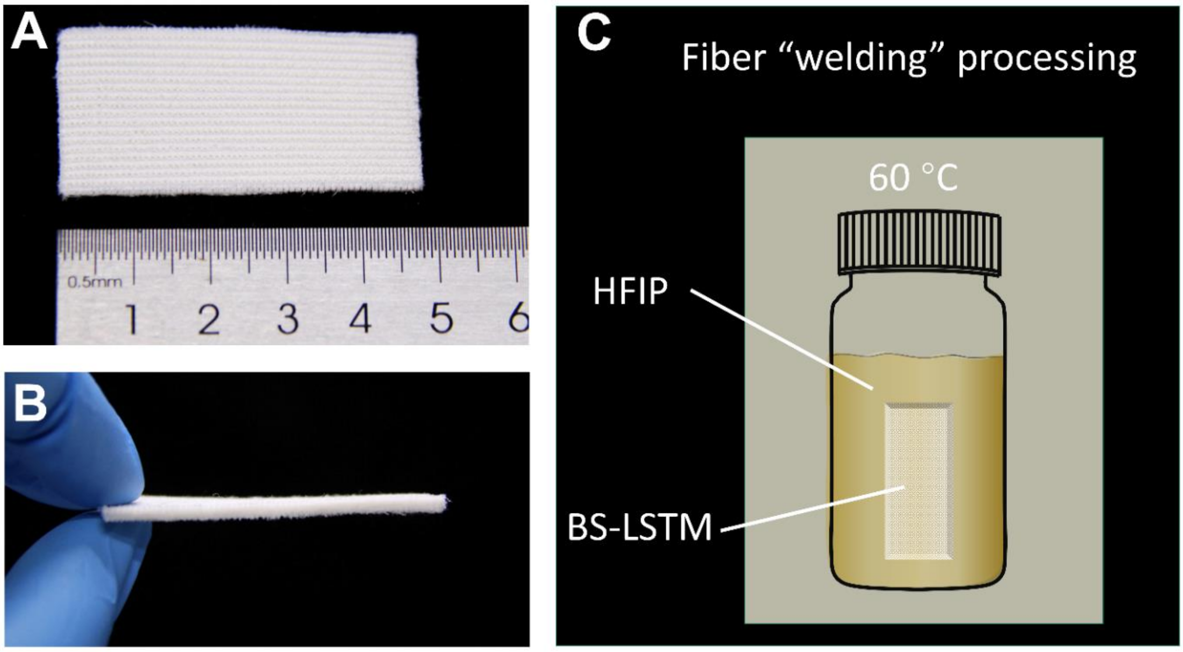

Figure S2. A) Photograph of the surface of BS-LSTM (laminated structure). B) Photograph of the cross-section of BS-LSTM (laminated structure). C) Schematic of the setup of the fiber "welding" processing.

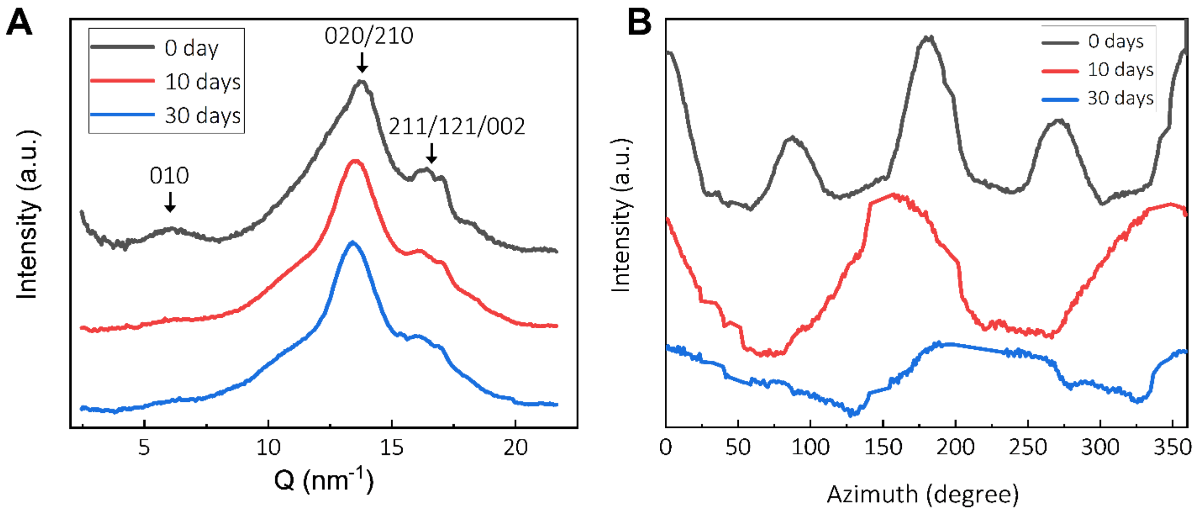

Figure S3. Radial A) and azimuthal B) WAXS profiles of the silk fabric after the "welding" process for 0, 10 and 30 days. The scattering vector Q is averaged in the ca. 10–15 nm $^{-1}$ range.

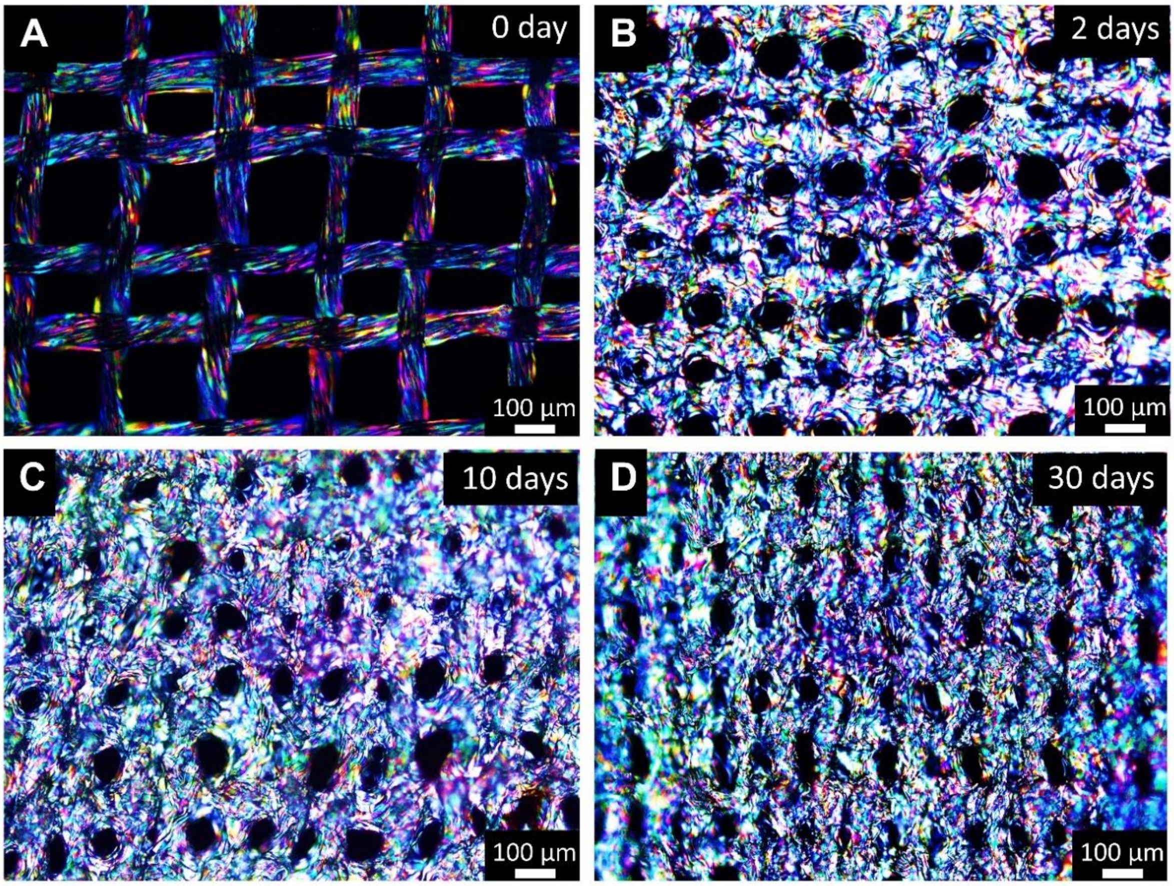

Figure S4. Polarizing microscopy images of silk fabric after the "welding" processing for 0, 2, 10, and 30 days, respectively.

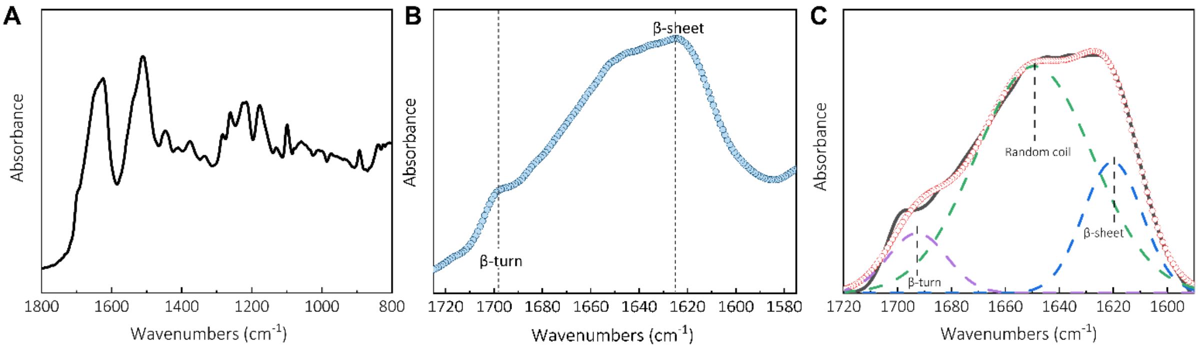

Figure S5. FTIR spectrum of dissolved silk fibroin after drying at 60 °C for two days in the range of amide band A), and amide I band B). C) The deconvolution results of the amide I band.

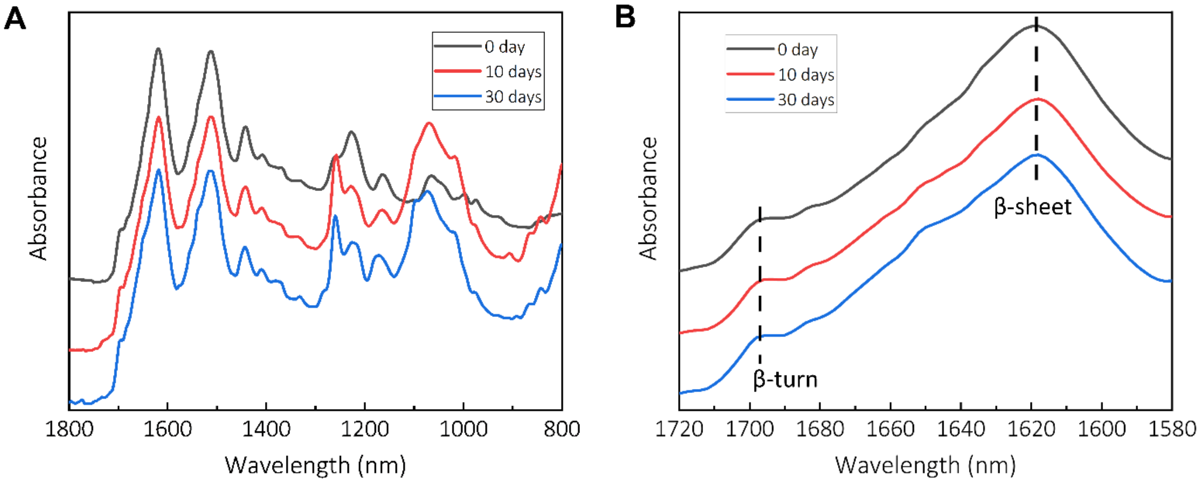

Figure S6. A) FTIR spectra of silk fabric after the "welding" processing for 0, 2, 10, and 30 days, respectively. B) FTIR spectra of silk fabric in the amide I band.

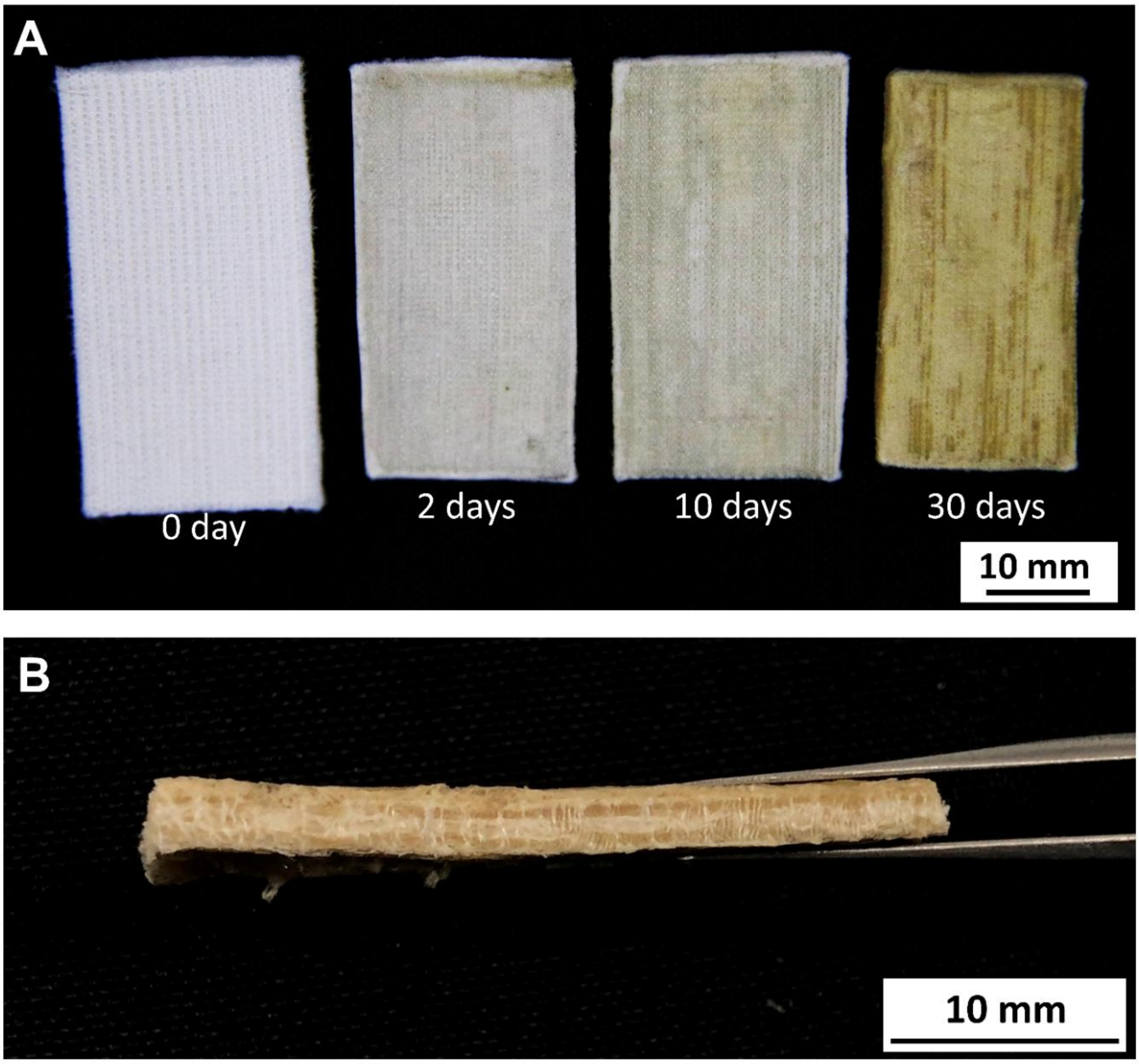

Figure S7. A) Photographs of BS-LSTM after the "welding" processing for 0, 2, 10, and 30 days, respectively. B) Photograph of the cross-section of the BS-LSTM after the "welding" processing for 30 days.

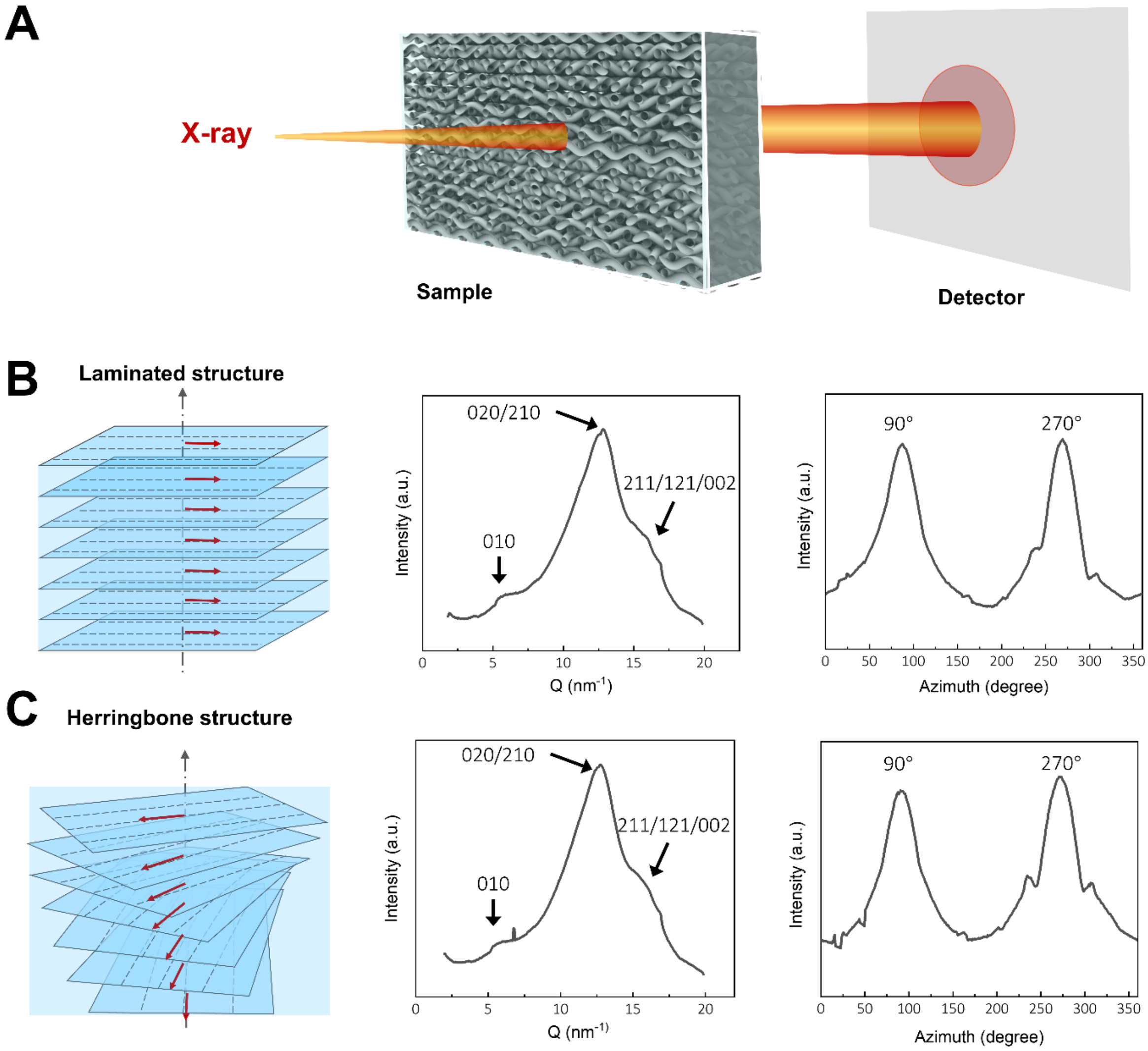

Figure S8. A) Schematic illustration of the in-plane X-ray exposure geometry. B) Radial (middle) and azimuthal (right) WAXS profiles of the laminated structure after the "welding" process for 10 days. C) Radial (middle) and azimuthal (right) WAXS profiles of the herringbone structure after the "welding" process for 10 days. In B) and C), the scattering vector Q is averaged in the ca. 10–15 nm $^{-1}$ range.

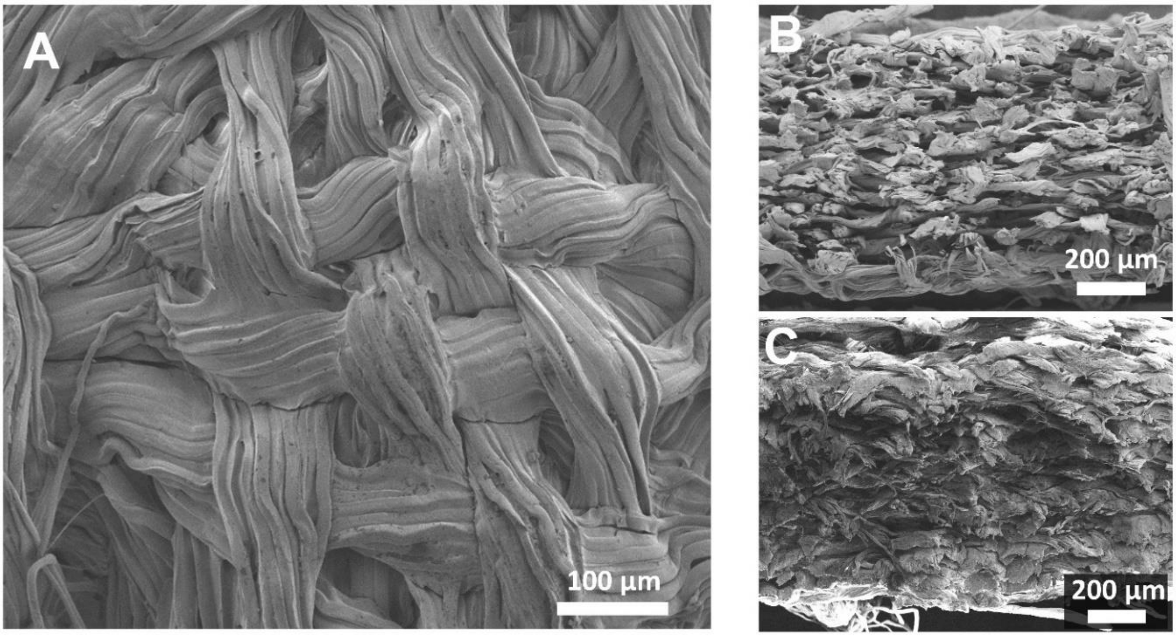

Figure S9. A) SEM image of the surface of the BS-LSTM after "welding" processing for 10 days. B) and C) SEM image of the cross-section of the laminated structure B) and herringbone structure C) after the "welding" process for 10 days.

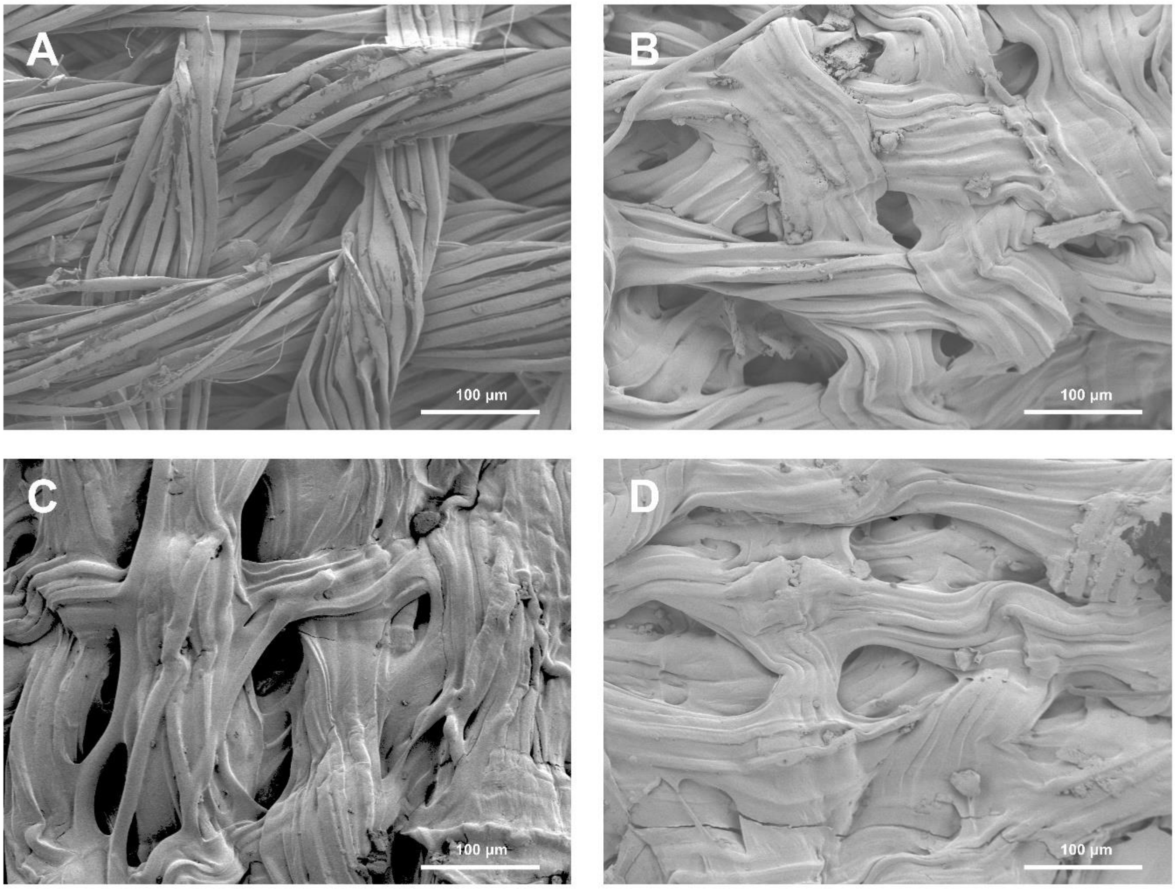

Figure S10. SEM image of the surface of the BS-LSTM after "welding" processing for 0 days A), 2 days B), 10 days C), and 30 days D).

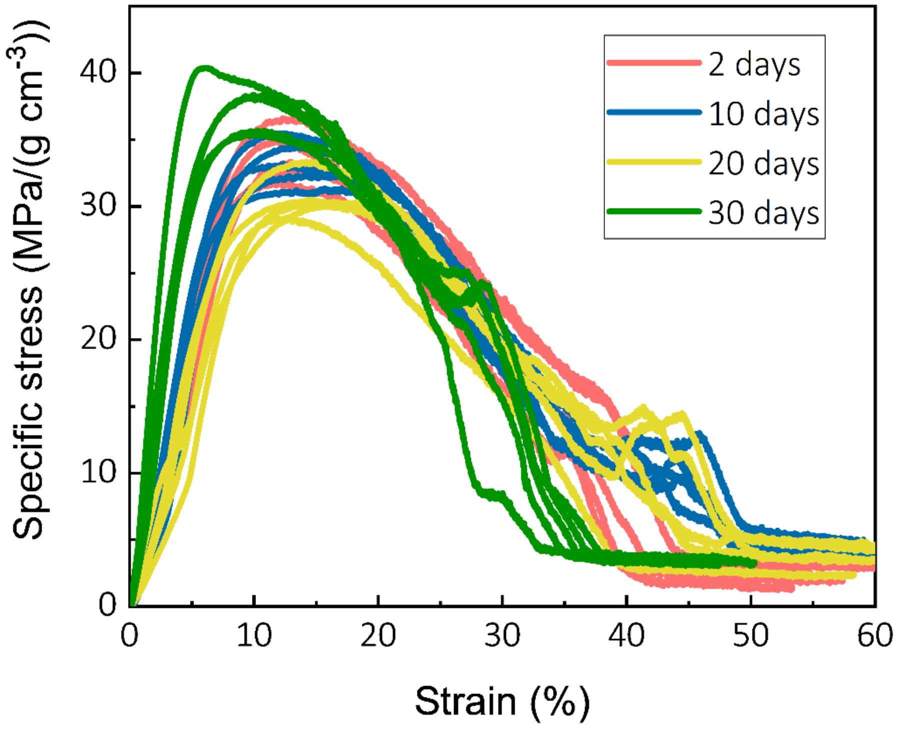

Figure S11. Specific stress-strain curves of BS-LSTM (herringbone structure) after the "welding" processing for different periods.

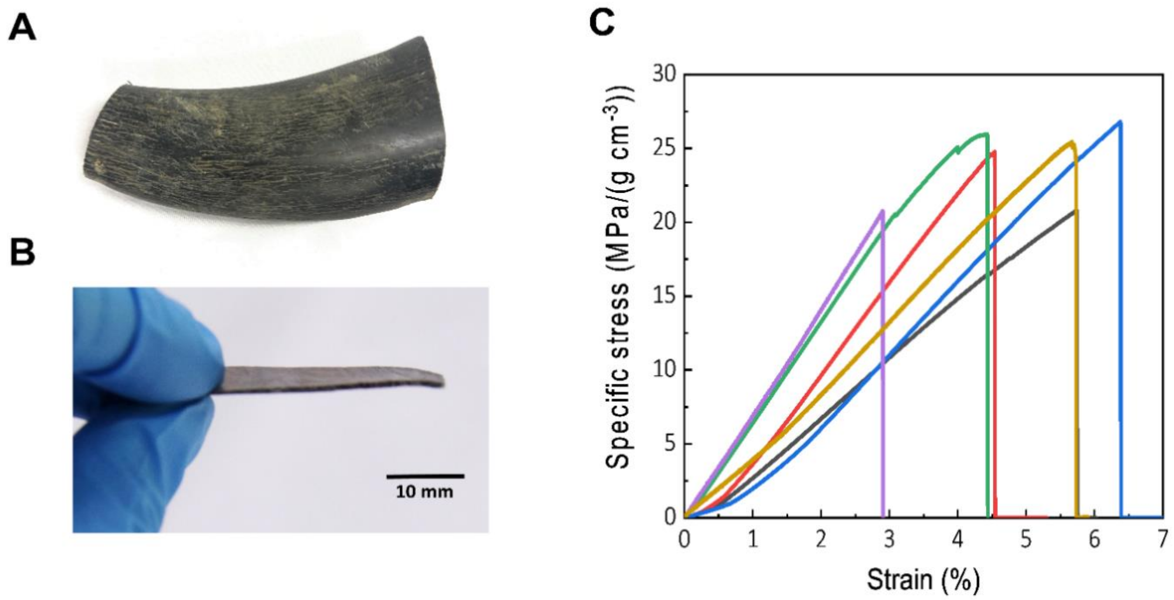

Figure S12. A) and B), Photograph of the buffalo horn A) and its section B) used for three bending tests. C) Specific stress-strain curves of buffalo horn sections.

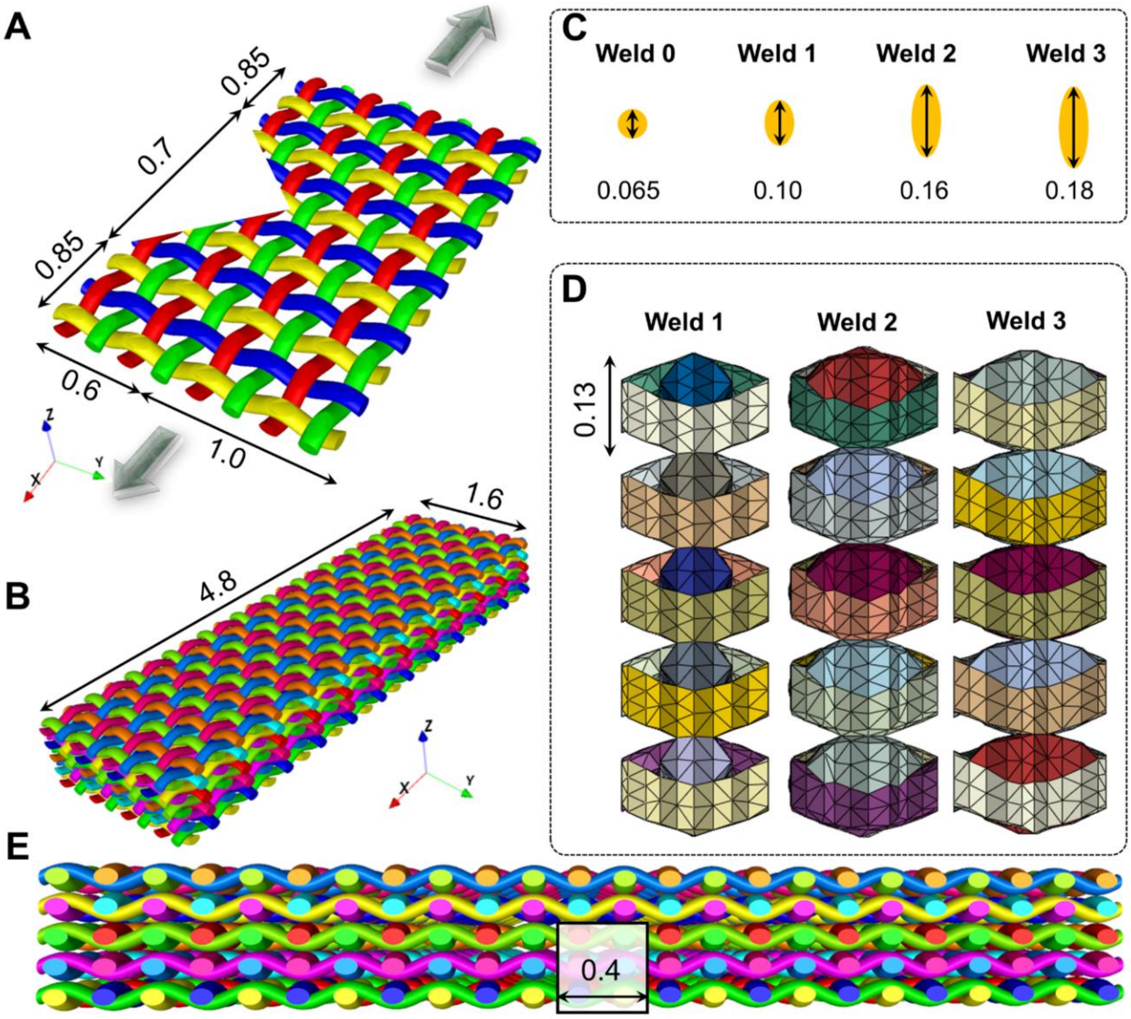

Figure S13. A) Model for tensile test. B) Model with laminate structure for three-bending test. C) Geometric parameters of the cross-sections of threads. D) Interactions between threads. E) Model with the artificial notch for three-bending test. (Unit is mm)

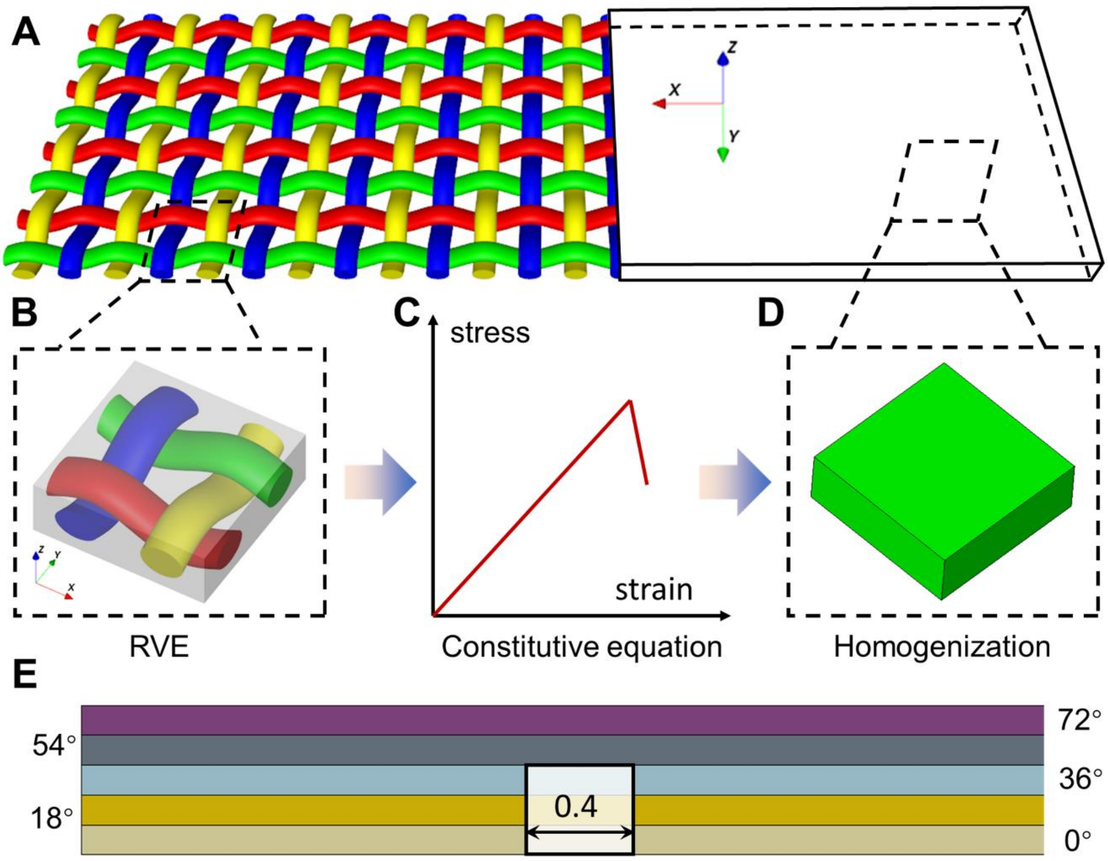

Figure S14. The schematic of homogenization method for the simulation of models with herringbone structure. A) The high-fidelity fabric sheet and its homogenization representation model. B) and C) shows the RVE model for calculating the homogenization mechanical properties. D) Homogenization representation model of RVE. E) Homogenization model for three-bending tests.

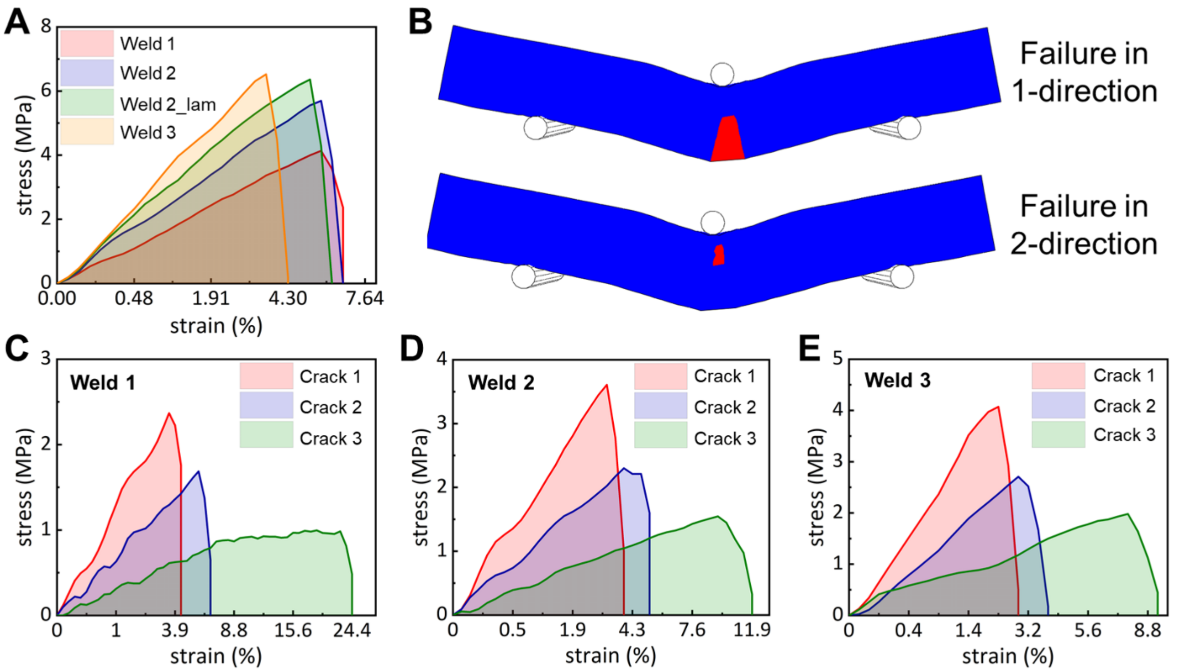

Figure S15. Simulation results of models with herringbone structures. A) The stress-strain curves of unnotched models at different welding degrees. B) The distribution of failures in models with herringbone structure at the welding degree of "Weld 2". C), D) and E) are the stress-strain curves of notched models at different welding degrees.

Table S1. Material properties of materials at different welding degrees (MPa).

	E_1	E_2 (E_3)	υ_{12} (υ_{13})	υ_{23}	G_{12} (G_{13})	G_{23}	X_T
Weld 0	2500	228	0.287	0.365	96	84	280
Weld 1, 2, 3	1625	228	0.287	0.365	62	84	182

Table S2. The homogenization properties of materials at different welding degrees (MPa).

	E_1	E_2	E_3	υ_{12}	υ_{13}	υ_{23}	G_{12}	G_{13}	G_{23}	X_T	ε_{X_T}
Weld 1	95.5	96	36.9	0.51	0.27	0.26	5.0	8.6	8.5	8.5	15.4%
Weld 2	202.7	202.5	17.4	0.43	0.34	0.33	20.7	10.9	10.8	11.9	12.0%
Weld 3	298.7	299	19.7	0.31	0.46	0.45	34.9	15.5	15.6	13.2	10.7%

Supplementary References

[1] G. D. Fang, B. Wang, J. Liang, *Compos. Sci. Technol*. **2019**, *181*, 107691.

[2] L. Zhao, T. L. Qin, J. Y. Zhang, R. A. Shenoi, *J. Compos. Mater*. **2013**, *47*, 2995.

[3] Z. Xia, Y. F. Zhang, F. Ellyin, *Int. J. Solids Struct*. **2003**, *40*, 1907.
